# Exploring racial differences in second-harmonic-generation–based prognostic indicators of metastasis in breast and colon cancer

**DOI:** 10.1117/1.BIOS.2.2.022703

**Published:** 2025-04-30

**Authors:** Tresa M. Elias, Danielle E. Desa, Edward B. Brown, Showmick Paul, Gabriel A. Ramirez, Bradley M. Turner, Kelley Madden, Raul S. Gonzalez, Anna Weiss, Edward B. Brown

**Affiliations:** a University of Rochester, Department of Biomedical Engineering, Rochester, New York, United States; b Morgridge Institute for Research, Madison, Wisconsin, United States; c University of Rochester, Department of Brain and Cognitive Sciences, Rochester, New York, United States; d University of Rochester Medical Center, Department of Orthopedics & Physical Performance, Rochester, New York, United States; e University of Rochester Medical Center, Department of Pathology and Laboratory Medicine, Rochester, New York, United States; f Brockport Research Institute, Brockport, New York, United States; g University of Rochester School of Medicine and Dentistry, Department of Surgery, Oncology, Rochester, New York, United States; h University of Rochester School of Medicine and Dentistry, Wilmot Cancer Institute, Rochester, New York, United States

**Keywords:** second-harmonic generation, multiphoton, cancer, prognostic, metastasis

## Abstract

**Significance:**

Second-harmonic generation (SHG) analysis of collagen internal structure and overall organization in the tumor microenvironment may enhance current metastasis prediction methods, which do not prognosticate with the same accuracy for patients of different races. For these optical tools to be clinically available, a multicenter trial is needed. We investigate if SHG-based prognostic signals vary with patient race, providing insight for designing such a trial.

**Aim:**

SHG imaging was performed on colon adenocarcinoma (CRC) and invasive ductal carcinoma (IDC) patient samples to derive two prognostic indicators. We assessed the association between these indicators and patient race.

**Approach:**

SHG images were analyzed as previously described to determine the forward- to backward-SHG scattering ratio (F/B) and fiber angle variability (FAV). Both prognostic measurements were compared between Black and White patients.

**Results:**

In the IDC cohort, F/B from the tumor–stroma interface differed significantly between demographic groups. For the CRC cohort, a trend was observed in the tumor–stroma interface and tumor bulk. FAV did not vary by race in either cohort.

**Conclusions:**

F/B variation with patient race suggests the relationship between F/B and metastatic outcome may vary with patient race. These findings highlight the potential need for race-specific prognostic algorithms to improve metastasis prediction for all patients.

Statement of Discovery We used second-harmonic generation imaging to quantify differences in collagen fiber internal structure and fiber angle orientation in the tumor microenvironment, both of which provide prognostic information in colon and breast cancer. Herein, we discover that these optical measurements vary with patient race, thus advancing the comprehensive and multifaceted prognostic tools.

## 1 Introduction

The discrepancy between cancer diagnosis, treatment, and overall survival between Black or African-American (BAA) and White or Caucasian-America (WCA) patients has been thoroughly documented.^1^ For example, although advances in detection and treatment have improved the 5-year survival for colorectal adenocarcinoma (CRC) patients since the mid-1970s, the 5-year survival for BAA patients (59%) remains lower than that of WCA patients (65%).^1^ Similarly for breast cancer, BAA patients have a lower 5-year survival for every stage of breast cancer compared with WCA patients, with the largest disparity in advanced stages (stage IV: 19% versus 30%, respectively).^1^

Studies have shown that 20% of CRC patients present with metastasis at the initial diagnosis and 25% of patients will experience a future metastasis.^2^ Among these patients, less than 20% will survive 5 years after their diagnosis.^2^ At least 30% of breast cancer survivors will metastasize^3^ and about one third of patients with metastatic breast cancer will survive 5 years.^4^ Alarmingly, studies have also shown that a common 21-gene chemotherapy guidance tool used to predict the likelihood of metastasis in breast cancer, Oncotype DX (ODX), does not predict chemotherapy benefit or prognosticate metastasis as accurately for BAA patients.^5^^–^^7^ Although this is a separate issue from the disparity in cancer mortality rates between BAA and WCA patients, and there are several possible explanations for these disparities, this finding still highlights a pressing demand to develop prognostic tools that perform accurately and effectively for patients of all ethnicities.

Although chemotherapy can improve outcomes in metastatic cancer, its side effects are toxic and can be fatal; in some cases, chemotherapy for breast cancer can cause secondary cancers such as acute myeloid leukemia.^8^ In CRC, chemotherapy has been observed to cause myelosuppression, a condition in which bone marrow produces fewer red and white blood cells,^9^ which can cause several other health problems for patients. Unfortunately, even with the latest prediction methods, a large percentage of treated patients may not have suffered metastases and were thus “overtreated,” whereas patients who are classified as low risk (and hence were untreated) often experience metastases; this has been observed in breast cancer^10^^–^^12^ and CRC.^13^^,^^14^ Hence, there is also a recognized need to improve the accuracy of metastatic prediction, to treat those who need it and spare those who do not.

Metastasis is a multi-step process that transports tumor cells from the primary tumor to a secondary location where they can grow and form a new tumor.^15^ The multi-step process of metastasis begins with the local invasion of tumor cells into the tumor microenvironment, which is an elaborate system of cells and blood vessels embedded in the extracellular matrix (ECM).^16^^,^^17^ In pursuit of novel prognostic tools, attention has been directed to optical signals from the ECM, which is composed of several proteins, including collagen.^18^ Collagen and cancer cells have an interesting bilateral relationship that encourages cancer progression; collagen properties can influence cancer cell behavior, whereas cancer cells can alter surrounding collagen fibers in a constructive^19^ and destructive^18^ manner. The physical properties of collagen in the ECM can initiate many physiological cascades, which can result in increased chances of tumor growth and progression. For example, an accumulation of collagen I can result in increased breast density, which multiplies the chances of breast cancer development;^20^^,^^21^ this increase in breast density also results in increased matrix stiffness, a known facilitator of cancer progression in breast^20^^,^^22^ and colorectal cancers.^23^^–^^25^

The internal structure of collagen is quantifiable with an intrinsic signal called second-harmonic generation (SHG), a nonlinear optical phenomenon that occurs when two photons scatter off a noncentrosymmetric material, producing a single photon with exactly twice the energy, and thus half the wavelength, of the incoming photons.^26^^–^^29^ SHG scattering directionality is affected by the internal structure of collagen fibers, including the diameter, spacing, and packing disorder of fibrils within fibers.^30^^–^^33^ We have previously demonstrated that the forward-to-backward scattered (where forward is in the direction of laser excitation) SHG signal (F/B), one measure of scattering directionality, is an independent prognostic indicator of overall and metastasis-free survival in estrogen-receptor positive (ER+) invasive ductal carcinoma (IDC) breast cancer and stage I CRC.^34^^–^^36^ In breast cancer, we specifically found this prognostic indicator to be far more effective when measured in the collagenous tumor–stroma interface than in the adjacent cellular tumor bulk.^36^ In addition, the orientation of collagen fibers relative to the tumor–stroma interface^37^^,^^38^ as well as the overall fiber angle variability (FAV) within the tumor bulk are also prognostic indicators of overall and disease-free survival in breast cancer.^39^^–^^42^ Although the prognostic abilities of FAV have yet to be studied in CRC, differences in fiber organization between healthy and diseased colon tissue have been observed.^43^^,^^44^

For a prognostic indicator such as F/B or FAV to be used in a clinical setting, the next logical step is a multicenter trial using samples from patients with known outcomes to train and test an algorithm that predicts patient outcomes and more effectively prescribes treatment. Unfortunately, there is tremendous underrepresentation of minorities in research cohorts,^45^^,^^46^ sometimes bringing the generalizability of trial results into question. In this study, we ask whether these prognostic optical measurements, F/B and FAV, vary between BAA and WCA patients in CRC and IDC cohorts to gain the necessary insight to plan such a trial.

## 2 Methods

### 2.1 Patient Samples

All patients were obtained from pathology archives at the University of Rochester Medical Center (URMC). These were patients with archived formalin-fixed paraffin-embedded (FFPE) tumor blocks, which were freshly cut into 5-μm sections and hematoxylin and eosin (H&E) stained by the URMC Department of Pathology. Recorded variables included the patient’s age at diagnosis, sex, race, BMI, and smoking history. Pathological variables included the pT stage and modified Bloom-Richardson grade. For both cohorts, grade 1 indicates a well-differentiated tumor, grade 2 indicates a moderately differentiated tumor, and grade 3 indicates a poorly differentiated tumor.^47^

#### 2.1.1 Colon adenocarcinoma

Eighty-eight patients with stage I colon adenocarcinoma were identified from the pathology archives at URMC from 2001 to 2012. All slides were validated by a trained pathologist, and their use was approved by the Institutional Review Board at the University of Rochester (IRB00001430). Pathologists categorized the patient’s pT stage based on whether the cancer had grown through the muscularis mucosa into the submucosa (pT1) and also grew into the muscularis propria (pT2).^48^

#### 2.1.2 Invasive ductal carcinoma

Two hundred forty-three patients with ER+ IDC were identified from the archives at URMC from 1999 to 2011. All patients were estrogen-receptor positive (ER+) and had been prescribed tamoxifen. All slides from this cohort were validated by a trained pathologist and their use was approved by the Institutional Review Board at the University of Rochester (IRB00001270). Pathologists categorized the patient’s pT stage based on the tumor’s size: pT1 indicates a primary tumor 2 cm or less in the greatest dimension, pT2 indicates a primary tumor between 2 and 5 cm in the greatest dimension, and pT3 indicates a primary tumor 5 cm or more in the greatest dimension.^48^ Microscopists were trained by pathologists to identify the clinically relevant regions of patient samples in both CRC and IDC cohorts.

### 2.2 Second-Harmonic Generation Imaging

SHG imaging was performed on samples typical of those already within the clinical workflow, specifically the FFPE H&E sections generated from primary tumor excisions. This facilitates the translation of these discoveries into clinically applicable methods and allows for easy identification of clinically relevant regions within the section. Therefore, we note that the reported F/B values are not necessarily the same as the F/B values that would be measured in unprocessed fresh tissue. It is also important to note when imaging thin samples on glass slides some of the backward-scattered photons could have been originally forward-scattered photons that were reflected by the glass side; similarly, some forward-scattered photons could be originally backward-scattered photons that were reflected by the glass coverslip.

#### 2.2.1 Colon adenocarcinoma

A Ti:Sapphire laser (MaiTai, Spectra Physics-MKS Instruments, Milpitas, California, United States) was circularly polarized at the sample using a Berek compensator to ensure equal excitation of all fiber orientations within the image plane, with 100 fs pulses at 80 MHz, 810-nm excitation wavelength. This was directed through an Olympus Fluoview FV300 scanner with a power of ∼8 to 10 mW at the sample. The laser was focused using a 20×, 0.95 NA water immersion objective (UMPLFL20XW, Olympus USA, Center Valley, Pennsylvania, United States), which also captured a backward-propagating SHG signal. The backward-propagating SHG signal was separated from the excitation beam using a 670-nm dichroic mirror, filtered (HQ405/30 m-2P, Chroma, Rockingham, Vermont, United States), and collected by a photomultiplier tube (H10492–003, Hamamatsu USA, Bridgewater, New Jersey, United States). The forward-scattered SHG was collected through an Olympus 0.9 NA condenser, reflected by a 565-nm dichroic mirror (565 DCSX, Chroma) to separate excitation light from the emission light, and captured using an identical filter (HQ405/30m-2P, Chroma) and identical photomultiplier tube. This produced an F image from the forward-propagating SHG light and a B image from the backward-propagating SHG light.

#### 2.2.2 Invasive ductal carcinoma

A Ti:Sapphire laser (Chameleon, Coherent Corp., Saxonburg, Pennsylvania, United States) was circularly polarized at the sample using a Berek compensator, with 100-fs pulses at 80 MHz, 810-nm excitation wavelength. This was directed through a Scientifica MP-2000 multiphoton scanner (Scientifica, Clarksburg, New Jersey, United States) with ∼8 to 10 mW at the sample. The laser was focused with a 20×, 0.95 NA water immersion objective (UMPLFL20XW, Olympus USA, Center Valley, Pennsylvania, United States), which also captured a backward-propagating SHG signal. This backward-propagating SHG signal was separated from the excitation beam using a 67-nm dichroic mirror, filtered (HQ405/30 m-2P, Chroma, Rockingham, VT), and collected by a photomultiplier tube (R0998U-210, Hamamatsu USA, Bridgewater, NJ). The forward-scattered SHG was collected through an Olympus 0.9 NA condenser, reflected by a 565-nm dichroic mirror (565 DCSX, Chroma) to separate excitation light from the emission light, and captured using an identical filter (HQ405/30m-2P, Chroma) and identical photomultiplier tube to produce F and B images.

**Fig. 1:**
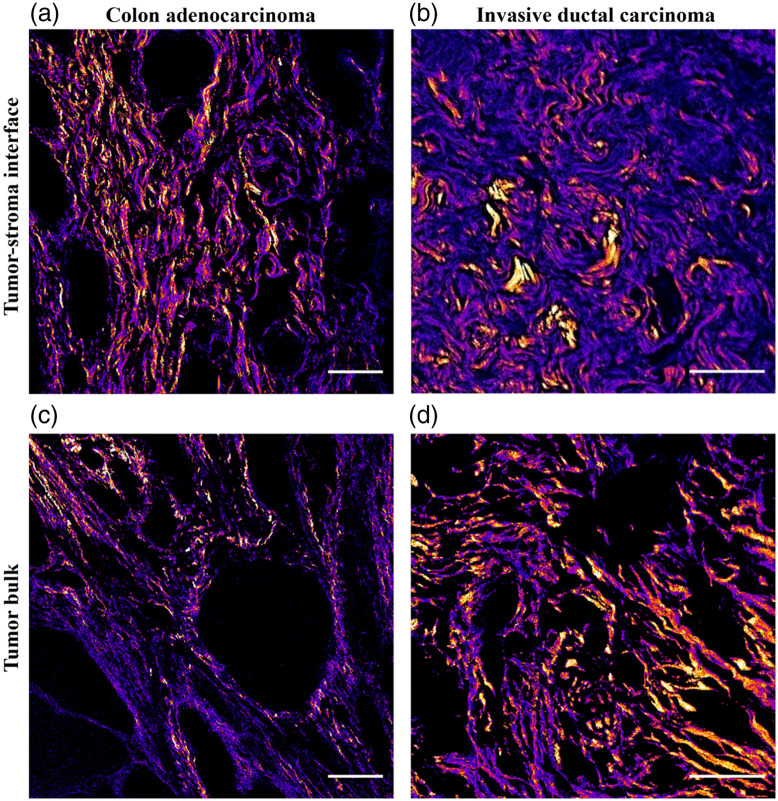
Representative second-harmonic generation images in primary resected tumors. Images were taken in the cellular tumor bulk and adjacent tumor–stromal interface in CRC and IDC patient samples. The F/B ratio was calculated for each field of view and is shown as a heatmap (lower F/B values are in purple) to illustrate differences between the regions. Scale bar=100  μm.

All images from both cohorts consisted of 512×512  pixels and were collected as z-stacks in 2  μm steps (3 slices, 6  μm total). Stacks were maximum intensity-projected to form a single F and single B image, which serves as a convenient pixel-by-pixel autofocus if the tissue is not perfectly parallel to the image plane. Three image pairs were taken in the cellular “tumor bulk” of each sample, followed by three pairs taken in the collagenous “tumor–stroma interface” directly adjacent to the tumor bulk (Fig. 1).

### 2.3 Image Analysis

#### 2.3.1 F/B analysis

To identify pixels within collagen fibers, images were thresholded as previously described.^36^ Briefly, images were binarized using a threshold of 0.6× the mean of the entire image. For each pixel, a series of progressively smaller windows were centered on the pixel and the percentage of non-zero pixels within the window was calculated. The smallest window size that produced more than 5% nonzero pixels was used for thresholding and applied to every pixel in the original non-binarized image. The pixel that the window is centered on receives a value of 1 if its value is greater than the mean pixel value within the window, and 0 otherwise. This algorithm was implemented in MATLAB (Mathworks, Inc., Natick, Massachusetts, United States) to produce a binary F and B mask, which were then multiplied together to produce an F/B mask and applied to the raw background-subtracted F/B image. The reported F/B value is obtained by taking the mean of the non-zero pixels in the final F/B image.

#### 2.3.2 Fiber organization

The overall variability in collagen fiber orientation (i.e., FAV) was quantified using a variation of the method of Dekker et al.^39^ CurveAlign was used to automatically identify fibers in an F image and their angles relative to a common direction,^49^^,^^50^ implemented in MATLAB (Mathworks, Inc., Natick, Massachusetts, United States). This automated method allows for more efficient identification of fibers than manual methods^51^ and will be more reproducible across users. The standard deviation of these angles was used as a measure of the relative organization of the collagen fibers in that image, with a lower standard deviation indicating lower FAV and hence more organized fiber ensembles.

### 2.4 Statistical Analyses

Unadjusted descriptives were compared using Mann-Whitney U and chi-squared tests for categorical and continuous variables, respectively. A mixed-effects regression model was estimated to compare F/B and FAV from the tumor–stroma interface and tumor bulk with a random effect at the patient level to account for the repeated measures of each slide. Statistical analyses were performed using Stata 16.1 (College Station, Texas, United States). P-values less than 0.05 were considered statistically significant.

## 3 Results

### 3.1 Patient Demographics

Table 1 shows the breakdown of demographics for the WCA and IDC breast cancer cohorts. For the CRC cohort, 77 (87.5%) patients were WCA and 11 (12.5%) were BAA. For the IDC cohort, 223 (91.8%) patients were WCA, whereas 20 (8.2%) patients were BAA. For both cohorts, it is important to note that not all patients had complete clinical or pathological data available, as indicated by the “N observed” rows of Table 1.

**Table 1:** Comparison across BAA and WCA patients of F/B, FAV, and clinical variables from CRC and IDC cohorts.

		CRC			IDC		
		WCA	BAA	P-value	WCA	BCA	P-value
	Total, N (%)	77 (87.50%)	11 (12.50%)	—	223 (91.77%)	20 (8.23%)	—
F/B in TSI	Mean (SD)	16.08 (4.36)	18.69 (3.74)	0.059	15.36 (5.71)	12.39 (5.59)	0.028
	Range (min, max)	(8.13, 26.66)	(13.51, 24.36)		(2.40, 29.77)	(4.70, 23.94)	
	N observed (%)	77 (100%)	11 (100%)		223 (100%)	20 (100%)	
F/B in TB	Mean (SD)	7.01 (3.33)	8.31 (2.55)	0.087	10.57 (4.83)	10.20 (5.57)	0.796
	Range (min, max)	(2.57, 20.62)	(3.80, 13.17)		(1.33, 29.75)	(1.27, 20.94)	
	N observed (%)	77 (100%)	11 (100%)		223 (100%)	20 (100%)	
FAV in TSI	Mean (SD)	39.18 (7.46)	40.49 (7.25)	0.549	46.03 (9.16)	46.76 (9.10)	0.700
	Range (min, max)	(22.56, 61.66)	(27.52, 50.93)		(24.60, 74.10)	(28.19, 63.81)	
	N observed (%)	77 (100%)	11 (100%)		223 (100%)	20 (100%)	
FAV in TB	Mean (SD)	44.42 (7.83)	43.95 (10.29)	0.815	47.23 (7.63)	45.92 (8.31)	0.548
	Range (min, max)	(27.79, 61.95)	(27.05, 64.25)		(19.54, 69.14)	(30.93, 60.65)	
	N observed (%)	77 (100%)	11 (100%)		223 (100%)	20 (100%)	
BMI	Mean (SD)	28.52 (4.95)	30.94 (6.52)	0.368	27.57 (5.88)	34.59 (6.69)	0.004
	Range (min, max)	(16.20, 39.20)	(25.00, 32.20)		(19.05, 54.10)	(25.75, 43.98)	
	N observed (%)	67 (87.01%)	9 (81.82%)		83 (37.22%)	8 (40.00%)	
Age at diagnosis	Mean (SD)	67.79 (13.03)	62.64 (8.59)	0.165	59.41 (12.96)	56.10 (11.25)	0.214
	Range (min, max)	(40, 93)	(52, 74)		(22, 91)	(43, 85)	
	N observed (%)	77 (100%)	11 (100%)		222 (99.55%)	20 (100%)	
pT stage	T1, N (%)	53 (68.83%)	8 (72.73%)	0.793	159 (71.30%)	15 (75.00%)	0.750
	T2, N (%)	24 (31.17%)	3 (27.27%)		51 (22.87%)	5 (25.00%)	
	T3, N (%)	0 (0%)	0 (0%)		6 (2.69%)	0 (0%)	
Grade	Grade 1, N (%)	16 (20.78%)	1 (9.09%)	0.367	91 (40.81%)	12 (60.00%)	0.283
	Grade 2, N (%)	46 (59.74%)	9 (81.82%)		86 (38.57%)	7 (35.00%)	
	Grade 3, N (%)	15 (19.48%)	1 (9.09%)		31 (13.90%)	1 (5.00%)	
History of smoking	Yes, N (%)	36 (46.75%)	4 (36.36%)	0.517	83 (37.22%)	11 (55.00%)	0.167
	No, N (%)	41 (53.25%)	7 (63.64%)		93 (41.70%)	6 (30.00%)	
Sex	Female, N (%)	38 (49.35%)	4 (36.36%)	0.420	223 (100%)	20 (100%)	—
	Male, N (%)	39 (50.65%)	7 (63.64%)		0 (0%)	0 (0%)	

We compared patient age, sex, smoking history, BMI, pT stage, and tumor grade between BAA and WCA patients (Table 1). In the CRC cohort, we observed no significant relationships between racial groups within any of the clinical variables. However, in the IDC cohort, we observed a significant relationship between BMI and patient race, which is consistent with existing studies.^52^^,^^53^ No other clinical variables were significant between BAA and WCA IDC patients.

### 3.2 F/B is Significantly Higher in Tumor–Stroma Interface of CRC and IDC Patient Samples

We have previously observed that there is a significant difference in F/B measured from the collagenous tumor–stroma interface and the adjacent cellular bulk of the tumor in other IDC patient cohorts.^36^ Representative F/B images from both areas in each tumor type are shown in Fig. 1, where purple pixels indicate a lower F/B.

**Fig. 2:**
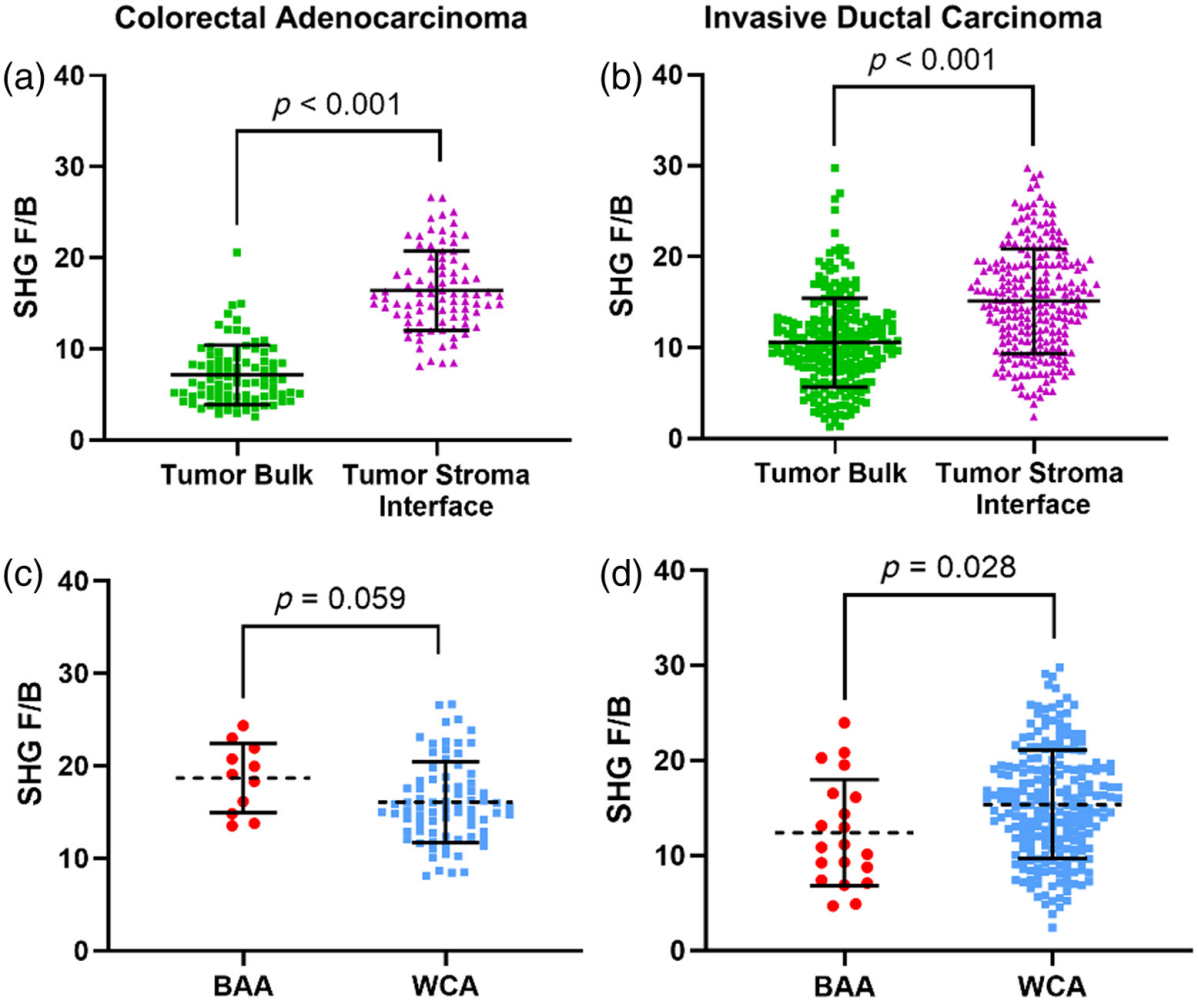
Collagen fiber internal structure measured by F/B in different tumor locations and its association with patient race. (a) F/B in CRC patients is significantly different when measured in the tumor bulk versus the tumor–stroma interface (7.17±3.26 and 16.41±4.35, respectively, n=88, p<0.001). (b) F/B in IDC is significantly different when measured in the tumor bulk versus the tumor–stroma interface (10.54±4.89 and 15.11±5.75, respectively, n=243, p<0.001). (c) F/B in CRCs trends toward being higher in BAA patients compared with WCA patients when measured in the tumor–stroma interface (14.3±2.51 (n=11) and 11.8±3.07 (n=77), p=0.059). (d) F/B in IDC patients is statistically significantly lower in BAA versus WCA patients when measured in the tumor–stroma interface (12.39±5.59 (n=20) and 15.36±5.71 (n=223), respectively, p=0.028). All values are reported as mean ± standard deviation.

In the CRC [Fig. 2(a)] and the IDC cohorts [Fig. 2(b)], we observed that F/B measured in the tumor–stroma interface was significantly higher than F/B measured in the tumor bulk (p<0.001 and p<0.001, respectively). These findings agree with previous studies of F/B in IDC^36^^,^^51^^,^^54^ and establish a similar trend in CRC.

### 3.3 F/B and Its Association with Patient Race

We next compared F/B from the tumor–stroma interface (i.e., the prognostic region in breast cancer^36^) and the tumor bulk between the BAA and WCA patients because further study is required to know which region F/B is most prognostic in CRC. F/B measured in the tumor–stroma interface in CRC patients reveals a trend toward a difference between BAA and WCA [Table 1, Fig. 2(c), p=0.059]. Interestingly, we also observed a trend in F/B between demographic groups in the tumor bulk (Table 1, p=0.087). We found that F/B measured in the tumor–stroma interface is significantly different between BAA and WCA IDC patients [Table 1, Fig. 2(d), p=0.028]. However, F/B from the tumor bulk does not differ between BAA and WCA patients (Table 1, p=0.796).

### 3.4 FAV from the Tumor–Stroma Interface Versus Tumor Bulk

**Fig. 3:**
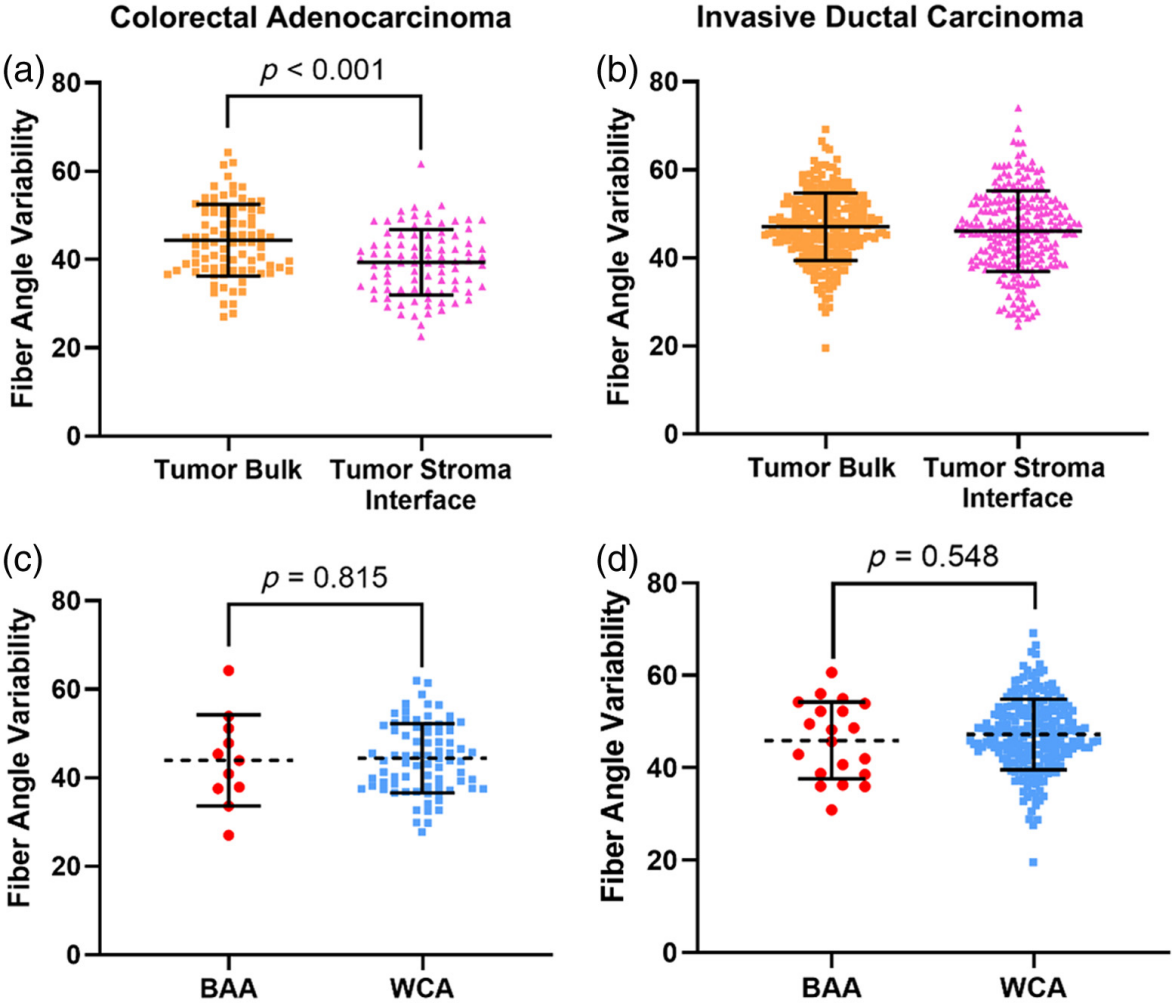
Collagen FAV measured in different tumor locations and its association with patient race. (a) FAV from the tumor bulk of CRC patients is significantly different from FAV from the tumor–stroma interface (44.36±8.11 and 39.5±7.41 respectively, n=88, p<0.001). (b) FAV from the tumor bulk of IDC patients is not significantly different from FAV from the tumor–stroma interface (47.12±7.68 and 46.09±9.137 respectively, n=243, p=0.118). (c) FAV measured from the tumor bulk of CRC patients does not vary significantly between BAA and CAA patients (43.95±10.29 (n=11) and 44.42±7.83 (n=77) respectively, p=0.815). (d) FAV measured from the tumor bulk of IDC patients does not vary significantly between BAA and WCA patients (45.92±8.31 (n=20) and 47.23±7.63 (n=223), respectively, p=0.548).

We next measured FAV using the same SHG images. FAV measured in the tumor–stroma interface of CRC was significantly lower than FAV measured in the tumor bulk [Fig. 3(a), p<0.001]. However, FAV measured in the tumor–stroma interface of IDC was not significantly different than FAV measured in the tumor bulk [Fig. 3(b), p=0.118]. The results from the IDC cohort agree with previous studies that found FAV to be similar when measured in the tumor bulk and tumor–stroma interface^41^; no previous studies have yet compared FAV from the tumor bulk and tumor–stroma interface in colon cancer.

### 3.5 FAV from the Tumor Bulk and Its Relationship to Patient Race

We compared FAV measured in the tumor bulk (where FAV is prognostic in breast cancer^39^^,^^41^) between BAA and WCA patients. FAV was not significantly different between BAA and WCA patients in either the CRC [Fig. 3(c), p=0.815] or IDC cohort [Fig. 3(d), p=0.548].

## 4 Discussion

F/B is a prognostic indicator of metastasis-free survival time in breast cancer^34^^,^^36^^,^^39^^–^^41^ and colon cancer.^34^ Although FAV has only been shown to be a prognostic indicator of metastasis-free survival time in breast cancer,^39^^–^^41^ recent CRC studies have revealed significant differences in fiber organization between healthy and diseased tissue^43^^,^^44^ and between different tumor grades.^44^ With further study of patient outcomes, FAV has the potential to also be a prognostic tool for CRC. For these tools to become clinically useful in predicting metastatic outcomes for patients, the next step would be to perform a multi-center trial where primary tumor excisions are imaged, F/B and FAV are determined, and follow-up data are secured and compared with these metrics. Training and validation subsets would then be used to derive and test a prognostic algorithm. Because minority groups are typically underrepresented in research cohorts,^45^^,^^46^ this multi-center trial must be designed carefully to cater to all ethnicities. The socioeconomic status of patients should also be considered when designing this future trial because lower socioeconomic status is also correlated with poorer cancer survival and mortality.^55^ Lower socioeconomic status is also associated with smoking, abuse of alcohol,^56^^,^^57^ and exposure to carcinogens and pathogens,^58^^,^^59^ all of which are risk factors for cancer.^55^ In addition, we know that ODX does not predict chemotherapy benefit or prognosticate as accurately for BAA patients in breast cancer.^5^^–^^7^ Consequently, any prognostic method developed without appropriately addressing this disparity runs the risk of only serving part of the patient population. To better understand the impact of race on these two prognostic indicators, we quantified the relationship between each indicator and patient race, as well as several other clinical variables when available.

We first confirmed a significant difference in F/B measured in the tumor bulk versus the tumor–stroma interface of IDC patients [Fig. 2(a), p<0.001], in line with previous measurements in other IDC cohorts.^36^^,^^51^^,^^54^ In the tumor–stroma interface, where F/B is prognostic in breast cancer, we found a significant difference between F/B measured in BAA and WCA patients and that BAA IDC patients had a lower F/B compared with WCA patients [Fig. 2(d), p=0.028]. Previous studies, in a different cohort where race was not recorded, have demonstrated that IDC patients with low F/B from the tumor–stroma interface have a worse chance of metastasis-free survival.^36^ Thus, for the IDC cohort, the BAA patients are displaying a more metastatic phenotype.

In the CRC cohort, our results suggest a trend in F/B between BAA and WCA patients when measured in both the tumor–stroma interface and the tumor bulk (Table 1, p=0.059 and p=0.087, respectively). Although we have previously demonstrated that F/B is most prognostic in the tumor–stroma interface relative to tumor bulk in breast cancer, no such study has yet been performed in colon cancer. This result suggests that wherever F/B proves to be most prognostic in colon cancer, there is a trend toward BAA patients having a higher F/B value relative to their WCA counterparts. Previous studies (in a CRC cohort where race was not recorded) demonstrated that patients with a higher F/B had a shorter disease-free survival time.^34^ Although further study and increased sample size are warranted, the data here suggest BAA patients in the CRC cohort display a more metastatic phenotype than WCA patients.

Previous studies have shown that IDCs with lower F/B values and CRCs with higher F/B values have poorer metastatic outcomes. In these cohorts, a trend is observed of BAA patients having a higher tumor–stromal and tumor bulk F/B than WCA in CRCs and a lower tumor–stroma F/B in IDCs. Consequently, our results indicate that in both cohorts these BAA patients have an F/B value consistent with more metastatic phenotype relative to the WCA patients. This is consistent with literature evidence of similar demographic differences in other tumor properties including gene expression, microvessel density, immune cell count, vitronectin, and distribution of tumor subtypes.^60^^–^^62^

Within the IDC cohort, we observed a statistically significant difference in BMI between BAA and WCA patients (Table 1, p=0.004). Although this is consistent with existing literature,^52^^,^^53^ this result leads one to ask if there is a relationship between patient BMI and F/B irrespective of race. To investigate this, all patients in the IDC cohort were divided into three groups based on BMI; BMI less than 25 is considered normal, BMI between 25 and 30 is considered overweight, and BMI over 30 is considered obese. F/B from the prognostic tumor–stroma interface was compared across these BMI categories using a Kruskal-Wallis test; no statistically significant difference was found (Table 2 in the Appendix, p=0.606).

**Table 2:** Comparison of F/B from the tumor–stroma interface across patient BMI, irrespective of race, in the IDC cohort.

	Normal (<25)	Overweight (≥25, <30)	Obese (≥30)	Total	P-value
TSI F/B, Mean (SD)	14.3 (5.5)	15.0 (6.2)	13.4 (6.0)	14.3 (5.9)	0.606
N	28	37	26	91	

If F/B varies with race, it is also possible that the relationship between F/B and metastatic outcome also varies with race. If this is true, then training a prognostic algorithm with a random cohort of patients (who would be overwhelmingly WCA) would disproportionately yield inaccurate predictions for demographics other than WCA patients. Therefore, it should be a priority to recruit enough BAA patients for this future research cohort to develop a prognostic algorithm that equally serves both demographics. If it is found that the relationship between F/B and metastatic outcome does vary with race, then different prognostic algorithms can be developed for different demographic groups.

We also measured FAV in both cohorts, which when measured in tumor bulk has been shown to be correlated with lymph node metastasis^39^ and metastatic outcomes^40^^,^^41^ in breast cancer. It is important to note that FAV is not the same as “tumor-associated collagen signature 3,” otherwise known as TACS-3, which is a measure of the tendency of collagen fibers at the tumor boundary to lie perpendicular to the identified edge and is also prognostic of metastatic outcome.^37^^,^^38^ We tested a second hypothesis that FAV in the tumor bulk varies with patient race and found no statistically significant relationship in the CRC cohort [Fig. 3(c) and Table 1, p=0.815] nor the IDC cohort [Fig. 3(d) and Table 1, p=0.548]. We also observed similar results in the tumor–stroma interface for the CRC cohort (Table 1, p=0.549) and the IDC cohort (Table 1, p=0.700). Although we have discussed the importance of diversity in research cohorts, this particular result suggests that patient race is likely not a significant factor in the future development of prognostic algorithms that exploit collagen FAV.

## 5 Conclusions

F/B is prognostic of metastasis-free survival in IDC breast cancer and stage I colon adenocarcinoma and is most prognostic in the breast when measured in the tumor–stroma interface. FAV is a prognostic indicator of overall and disease-free survival in IDC when measured in tumor bulk and has the potential to offer prognostic information in CRC. SHG imaging was used to quantify F/B and FAV in a cohort of 88 CRC patients and 243 IDC patients. In both cohorts, F/B measured from the tumor–stroma interface varies with patient race. This result poses the possibility that the relationship between F/B and metastatic outcome may also vary with race, thus highlighting the importance of recruiting a heterogeneous cohort of patients in a future multi-center trial that aims to use F/B to develop a prognostic algorithm. FAV has been shown to have prognostic capabilities when measured in the tumor bulk and could potentially be another variable in this future prognostic algorithm. In both cohorts, we found no statistically significant relationship between FAV and race. Although patient demographics should always be considered when designing research cohorts, this result suggests it is less pertinent for acquiring accurate predictions from FAV data.

## 6 Appendix: Supplementary Table

## Data Availability

MATLAB code for adaptive thresholding and computing F/B is available on CodeOcean: https://doi.org/10.24433/CO.6271963.v1.
