# Supplementary material for: Exploring racial differences in second-harmonic-generation–based prognostic indicators of metastasis in breast and colon cancer
Source: Biophotonics Discov. Author manuscript; Available in PMC 2025 Aug 22. (PMC12369920; doi:10.1117/1.BIOS.2.2.022703)
Supplement: Supplementary file 1 [file NIHMS2089927-supplement-1.pdf]

6 Appendix: Supplementary Table

Table 2 Comparison of F/B from the tumor–stroma interface across patient BMI, irrespective of race, in the IDC cohort.

	Normal (<25)	Overweight (≥25, <30)	Obese (≥30)	Total	*P*-value
TSI F/B, Mean (SD)	14.3 (5.5)	15.0 (6.2)	13.4 (6.0)	14.3 (5.9)	0.606
N	28	37	26	91	
